# Polycarboxylate Functionalized Graphene/S Composite Cathodes and Modified Cathode-Facing Side Coated Separators for Advanced Lithium-Sulfur Batteries

**DOI:** 10.1186/s11671-019-3099-3

**Published:** 2019-08-05

**Authors:** Maryam Sadat Kiai, Omer Eroglu, Huseyin Kizil

**Affiliations:** 1 Nano-Science and Nano-Engineering Program, Graduate School of Science, Engineering and Technology, Istanbul Technical University, 34469 Istanbul, Turkey; 2 Metallurgical and Materials Engineering Department, Istanbul Technical University, 34469 Istanbul, Turkey

**Keywords:** Lithium-sulfur battery, Polycarboxylate functionalized graphene cathode, Sulfur host material, Polysulfides

## Abstract

Sulfur-hosting novel materials as a cathode for lithium-sulfur batteries are in the focus of many research to enhance the specific capacity and cycling stability. Herein, we developed composite cathodes consisting of polycarboxylate functionalized graphene (PC-FGF) doped with TiO_2_ nanoparticles or poly1,5-diaminoanthraquinone (PDAAQ) and sulfur to enhance chemisorption property toward polysulfides. Additionally, PC-FGF/sulfur composite cathode functions as an efficient trapping site for polysulfides spices as well as contributes to facilitate electron and Li-ions movement toward or from the cathode. In the first experiment, the cell with sulfur incorporated TiO_2_/PC-FGF cathode is assembled with three different cathode-facing side-coated glass fiber separators. At the second test, PDAAQ/PC-FGF cathode is assembled with the same separator materials as before.

The best electrochemical performance observed was sulfur incorporated TiO_2_/PC-FGF cathode with PDAAQ/PC-FGF-coated separator having a high discharge capacity of 1100 mAh g^− 1^ at 0.5 C after 100 cycles. It is found that the combination of TiO_2_/PC-FGF/sulfur cathode and PDAAQ/PC-FCF separator could serve as promising cathode and separator material due to high cycling stability and rate capability for advanced Li-S batteries.

## Background

Lithium-sulfur batteries with high energy density (~ 2600 Wh kg^− 1^) and high theoretical specific capacity (1672 mAh g^− 1^) are taken into consideration for large-scale energy applications. In fact, the application of lighter Li-S cell with long-cycle life and fast charge discharge-regime is expected to be commercialized in moderate to high-volume market [1]. Due to high abundance and non-toxic nature of sulfur, Li-S battery system satisfies both cost-effective and environmental considerations. Despite the aforementioned advantages, there are several limitations with Li-S technology which cause a gap between the theoretical and practical energy density of Li-S battery. The major problems are related to very complex reaction mechanisms of lithium and sulfur. The reaction of S_8_ with Li^+^ during the discharge process leads to the formation of soluble polysulfides in the electrolyte and their diffusion toward the anode. Diffusion of these active spices with insulating nature causes the shuttle effect, which is known as the most serious problem about capacity degradation in the Li-S cells. Diffusion of soluble polysulfides toward the anode corrodes the lithium anode, suppresses ion mobility, and gives rise to the loss of active material [2, 3].

Nanocomposite has become the most attractive cathode material due to its efficiency, low cost, stability, and high electrical conductivity [4, 5]. Also, they can serve as electron collector and transporter resulting in the increase of ion and electron mobility [6].

Sulfur-carbon nanocomposites with micro-/meso-porous structural design were investigated recently to encapsulate sulfur into porous substrates [7–10]. Nanocomposite carbon host cathode with high electrical conductivity contributes to enhance redox reaction and improves sulfur and other polysulfides products adsorption on the surface of carbon host. CNFs, CNTs, and graphene are known as the most popular carbon host materials [11–14].

Among carbonous host materials, graphene-based hosts with reasonable electrical conductivity and admiring flexibility and mechanical strength have been investigated due to their enhancement capability of electrochemical reactivity of sulfur and overall cycle life.

Several studies have been investigated on the chemical interactions between the functional group (e.g., oxygen group or hydroxyl group) on graphene and polysulfides to enhance the polysulfides immobilization [15, 16]. In the study of Wang et al., nitrogen-doped graphene nanosheets/sulfur (NGNSs/S) composite were investigated as a conductive host to entrap S/polysulfides in the cathode part. The NGNSs/S composite delivered an initial discharge capacity of 856.7 mAh g^− 1^ and a reversible capacity of 319.3 mAh g^− 1^ at 0.1 C [17].

Polymer/graphene hybrid porous electrodes have also been investigated recently as promising electrode materials for lightweight and flexible energy storage devices with stable high performance [18, 19].

Polyaniline (PANI)-modified cetyltrimethylammonium bromide (CTAB)–graphene oxide (GO)–sulfur nanocomposites exhibited significant enhancement in the performance of lithium-sulfur batteries. CTAB-coated sulfur layer is believed to be able to trap polysulfides and alleviate dissolution of polysulfides through the formation of Li_2_S_x_ … N. The cell showed the capacity of 970 mAh g^−1^ at 0.2 C and retained it at 715 mAh g^− 1^ after 300 cycles, also initial capacity was 820 mAh g^−1^ at 0.5 C with a capacity retention of 670 mAh g^−1^ after 500 cycles [20].

The quinone polymers such as poly 1, 5-diamino-anthraquinone (PDAAQ) are another promising additives for graphene hybrid cathodes and separators. They involve two-electron redox reactions. Combination of quinone polymers with excellent conductive carbon materials, such as porous carbon materials, graphene, and CNTs, improve the electronic conductivity of the quinone materials and enhance rate capabilities and cycling performances. Additionally, conductive carbon promotes the utilization of the quinone-active materials during charge/discharge process [21]. This novel structure also increases the active specific surface area by creating additional active sites [22].

Recently, application of PDAAQ-potassium functionalized graphene nanoplates (K-FGF) in the separator of Li-S battery was introduced by Kizil et al. to enhance the electrochemical performance of Li-S battery. They reported PDAAQ-K-FGF (cathode and anode-facing side) coated separator delivered reversible capacities of 1001 and 776 mAh g^− 1^ at 0.5 C and 1 C with Coulombic efficiency as high as 99%. They found K-FGF with high hydrophilicity could increase the electrochemical performance by decreasing the internal resistance [23].

Metal oxides in graphene/S hybrid cathode are used as promising additives to the sulfur cathode. Mostly used additives of metal oxides are listed as manganese oxide, nickel oxide, alumina, silica, and titania [24–28].

Hue et al. reported new mesoporous TiO_2_/reduced graphene oxide (rGO) as an efficient polysulfide trapping in the cathode. TiO_2_@rGO hybrid structure has been shown to trap polysulfide products effectively by means of strong chemical bonding with oxygen double bonds. Also, incorporating GO in the cathode enhanced the electrical conductivity and improved polysulfides trapping ability providing large surface area. They found that incorporation of TiO_2_@rGO in the cathode exhibits capacities of 1116 and 831 mAh g^−1^ at the current densities of 0.2 C and 1 C (1 C = 1675 mA g^− 1^) after 100 and 200 cycles [29].

Herein, we have investigated the influence of different dopants incorporated into the sulfur-polycarboxylate functionalized graphene (PC-FGF) cathode. In the first test, TiO_2_ nanoparticles are added into PC-FGF/S cathodes to enhance active material reutilization. Also, TiO_2_ can effectively trap soluble polysulfides both at the cathode and the separator [30, 31] and increase the specific capacity of the lithium-sulfur battery. In the second experiment, PDAAQ is added into PC-FGF/S cathodes to improve chemical binding between the polysulfides and quinone group of PDAAQ and enhance redox reactions. The cells with these cathodes and three different coatings of PDAAQ/PC-FGF, PDAAQ/CTAB/PC-FGF, and PC-FGF/TiO_2_/MWCNT on the cathode-facing side of the glass separators were tested. Conversion and physical confinement of polysulfides at the cathode-separator interface, and the electrochemical performances of the cells were compared and discussed.

## Methods

### Materials and Chemicals

Polycarboxylate functionalized graphene (PC-FGF, Sigma-Aldrich), cetyltrimethyl- ammonium bromide (CTAB, BioXtra, ≥99% Sigma-Aldrich), 1, 5-poly diaminoanthraquinone (PDAAQ, technical grade, 85%, Sigma-Aldrich), titanium dioxide (TiO_2_, nanopowder rutile, 21 nm particle size, ≥ 99.5% trace metals basis, Sigma-Aldrich), multi wall carbon nanotube (MWCNT > 98% carbon basis, Sigma-Aldrich), sulfur (S, 99.5–100.5%, Sigma-Aldrich), bis (trifluoromethane) sulfonamide lithium (LiTFSI, 99.95% trace metals basis, Sigma-Aldrich), lithium nitrite (99.99% trace metals basis, Sigma-Aldrich), polyvinylidene fluoride (PVDF, Mw 1000–1200 kg/mol, Solef® 5130, Solvay), N-methyl-2 pyrrolidone (NMP, 99%, Sigma-Aldrich), 1,3-dioxolane (DOL, 99%, Sigma-Aldrich), and 1,2-dimethoxyethane (DME, 99.5%, Sigma-Aldrich) were used without any purification. Glass microfiber filters (Whatman, Grade GF/C) with 1.0 μm retention and circle size of 2.5 cm with the thickness of 260 μm were used as basis for coating.

### Separator Coating

For PDAAQ/CTAB/PC-FGF coating, a mixture of PDAAQ, CTAB, and PC-FGF (with a mass ratio of 2:2:1) was placed in an agate mortar and allowed to ground for 15 min to obtain the PDAAQ/CTAB/PC-FGF composite. Then, PVDF powder was added to this composite (with a mass ratio of 1:4) and ground until homogenous slurry was obtained. Subsequently, NMP solution (with a mass ratio of 1:9, contains 30% wt. PVDF) was added to the PDAAQ/CTAB/PC-FGF/PVDF mixture and allowed to stir for 30 min more to form a homogeneous slurry. The slurry was then coated on one side of a glass fiber separator and dried in an air oven at 60 °C for 2 h. PDAAQ/PC-FGF coating was prepared as described above. The only difference was related to PDAAQ/PC-FGF composite mass ratio which was defined as 1:1. For PC-FGF/TiO_2_/MWCNT coating, a mixture of TiO_2_, MWCNT, and PC-FGF (with a mass ratio of 2:2:1) was placed in an agate mortar and the same process was followed to obtain PC-FGF/TiO_2_/MWCNT-coated glass fiber separator. The thickness of uncoated glass fiber separator was 260 μm and the measured coating thickness of PDAAQ/PC-FGF, PDAAQ/PC-FGF/CTAB, and PC-FGF/TiO_2_/MWCNT were 53, 57, and 61 μm respectively.

### Cathode Preparation

To fabricate TiO_2_/PC-FGF/S cathode, the slurry with 60 wt% S, 15 wt% PC-FGF, 15 wt.% TiO_2_ nanopowder and 10 wt.% PVDF in NMP was coated onto an aluminum foil using doctor blade method. PDAAQ/PC-FGF/S cathode was prepared with 60 wt% S, 15 wt% PC-FGF, 15 wt.% PDAAQ, and 10 wt.% PVDF binder in NMP solvent and coated onto an aluminum foil using doctor blade method. (16 μm thickness, 1.32 cm^2^ in area). The coated cathode was dried in an air oven at 60 °C for 4 h. 1 M LiTFSI and 0.5 M LiNO_3_ in a solvent mixture of DME/DOL (1:1) was considered as an efficient electrolyte for polysulfides trapping. Amount of electrolyte in different coin cells was fixed at 20 μl/mg of S. The S loading set around 1.8–2.4 mg cm^−2^. The maximum S loading was 2.4 mg cm^−2^.

### Electrochemical Performance Characterization

The CR2032-type coin cells were assembled with TiO_2_/PC-FGF/S or PDAAQ/PC-FGF/S composite cathode, cathode-facing side coated separator, lithium metal anode, and electrolyte in an argon-filled glove box. The cells were cycled between 1.5 and 3 V on a Neware BTS 3008 battery tester at room temperature. Based on the sulfur mass in the cathode, specific capacities were determined. Surface characterization of modified cathodes and separators were carried out using SEM equipped with an EDS.

The cyclic voltammetry (CV) measurements were performed by Gamry Reference 600 with a scan rate of 0.2 mV s^−1^ in a potential range of 3–1.4 V (vs. Li^+^/Li). Electrochemical impedance spectroscopy (EIS) tests were performed by using Gamry Reference 600 to measure the internal resistance of the objected cells from 1 MHz to 1 Hz at an AC voltage amplitude of 10 mV.

## Results and Discussion

**Fig. 1:**
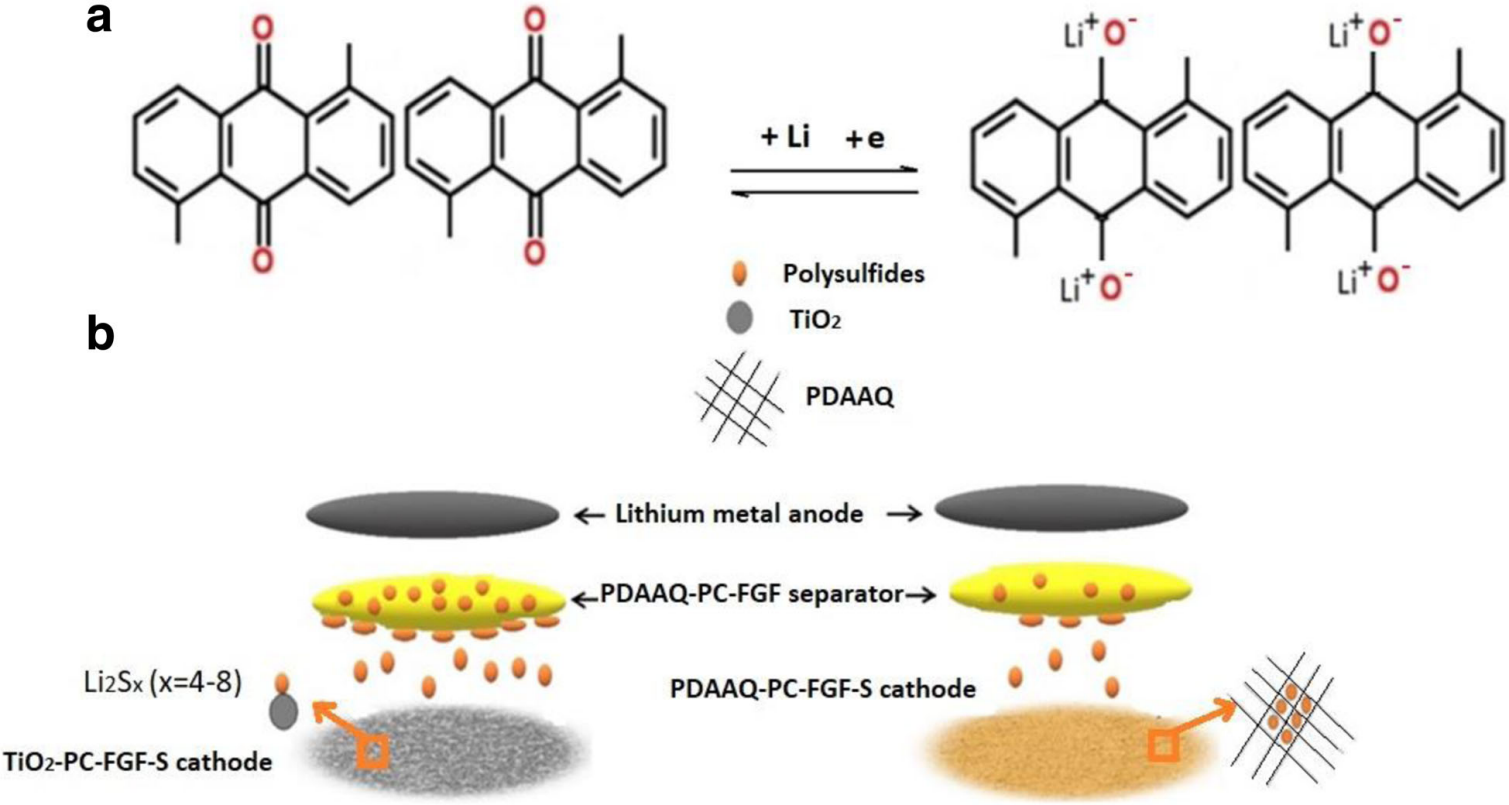
**a** Schematic diagram for the lithium ion insertion/deinsertion in the benzoquinone structures of PDAAQ. **b** Schematics of different polysulfide migration attitudes with two different cathodes

Schematics of Li-S cells with TiO_2_/PC-FGF/S or PDAAQ/PC-FGF/S cathode with PDAAQ/PC-FGF-coated glass fiber separator illustrate the behavior of polysulfide migration as shown in Fig. 1a, b.

As shown in Fig. 1a, during the charging process, one of carbonyl groups in PDAAQ accepts one electron and forms a free radical anion, and electrostatically adsorbs Li-ions. The redox mechanism of the PDAAQ is based on electron transfer reaction [21, 32].

As shown in Fig. 1b, PDAAQ/PC-FGF separator prevents the polysulfide diffusion into the anode side during charging and increases the deposition of polysulfides on the separator surface. PDAAQ with benzoquinone segments acts as a second current collector to confine intermediate polysulfides products into the cathode and enhances active material reutilization within the cathode compartment. PDAAQ with redox-active benzoquinone segments to PC-FGF separator proposed a novel separator with Li host capability through insertion and extraction of Li-ions in benzoquinone segments [33, 34]. As a cathode compartment, there is strong chemical adsorption between TiO_2_ nanoparticles at PC-FGF/S cathode and polysulfides. This novel cathode could suppress the dissolution of PS and prevent the shuttle effect by physical adsorption and chemical confinement [35].

**Fig. 2:**
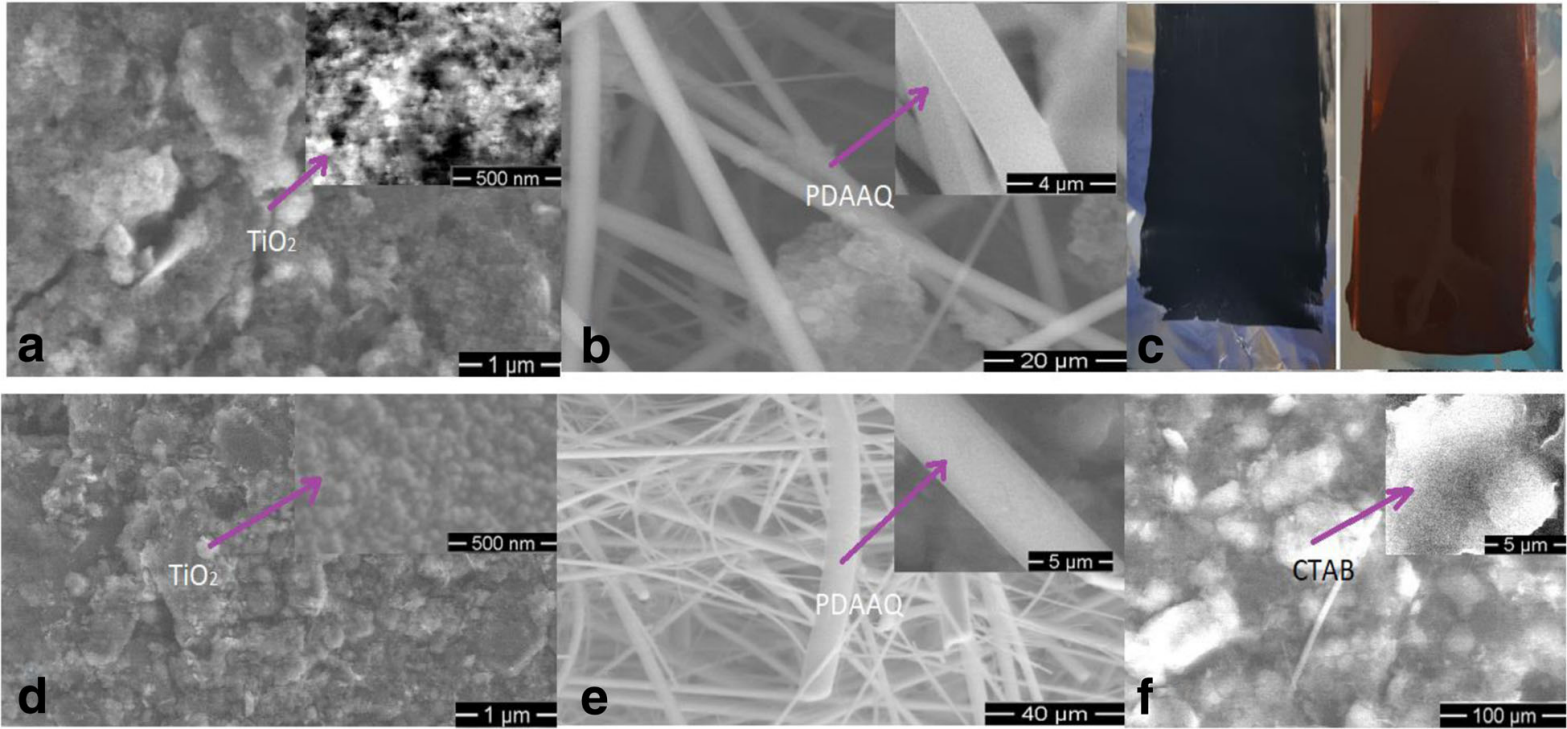
SEM images of **a** TiO_2_/PC-FGF/S cathode and **b** PDAAQ/PC-FGF/S cathode, the insets are high-resolution images. **c** Photographs of TiO_2_/PC-FGF/S (left) and PDAAQ/PC-FGF/S (right) cathode, SEM images of **d** TiO_2_/MWCNT/PC-FGF **e** PDAAQ/PC-FGF, **f** PDAAQ/CTAB/PC-FGF coated glass fiber separators, the insets are the high-resolution images

The SEM images of TiO_2_/PC-FGF/S and PDAAQ/PC-FGF/S cathodes are shown in Fig. 2a, b. Figure 2a shows that TiO_2_ particles are uniformly distributed into a continuous network of PC-FGF/S cathode. As shown in Fig. 2b, homogeneously dispersion of PDAAQ on the PC-FGF/S cathode provide sufficient sites to host active materials. Figure 2d–f represents the SEM image of TiO_2_/MWCNT/PC-FGF, PDAAQ/PC-FGF, and PDAAQ/CTAB/PC-FGF-coated glass fiber separators respectively. Uniform dispersion of TiO_2_ effectively confines the polysulfides within the separator and improves reutilization of intercepted active materials (Fig. 2d). Dispersion of PDAAQ on the PC-FGF-coated separator is similar to the dispersion of PDAAQ on the PC-FGF/S cathode and enhances porous sites to trap polysulfides (Fig. 2e).

**Fig. 3:**
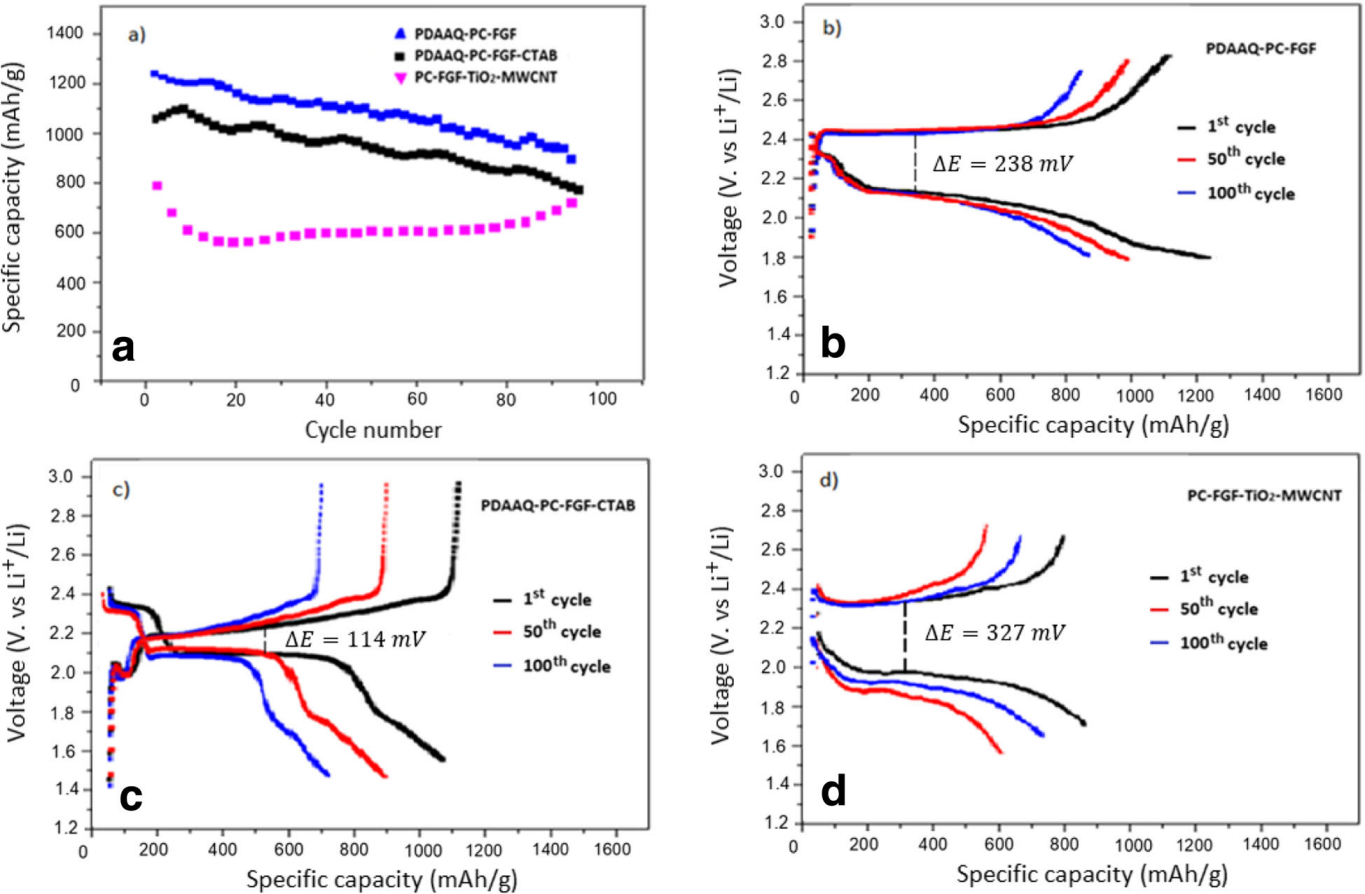
**a** Cycling performance of Li-S cells with PDAAQ/PC-FGF/S cathode and three different coated separators at current density of 0.5 C. **b** Galvanostatic charge/discharge profiles for the 1st, 5th, 100th cycles of Li-S cells with PDAAQ/PC-FGF, **c** PDAAQ/PC-FGF/CTAB, **d** PC-FGF/TiO_2_-MWCNT interlayers at 0.5 C with potential window of 1.5–3 V versus Li^+^/Li^0^

Figure 3a shows the cycling performance of Li-S cells with PDAAQ/PC-FGF/S cathode and three different coatings on the glass fiber separators. (1 C = 1672 mA g^−1^). It demonstrates that the cell with PDAAQ/PC-FGF coated separator exhibited the highest initial capacity of 1230 mAh g^−1^ at 0.5 C among others, and capacity retained at 900 mAh g^−1^ after 100 cycles. PDAAQ/PC-FGF/CTAB-coated separator showed initial capacity of 1040 mAh g^−1^ at 0.5 C with a capacity retention of 730 mAh g^−1^ after 100 cycles. It seems that when PDAAQ was applied both in the cathode and the separator, initial capacities were high with satisfactory discharge capacities between 1st and 100th cycles which were arising from quinone groups of PDAAQ with π-electrons which can undergo both interchain and intrachain movement in three dimensions [32].

As shown in Fig. 3a, the highest stability belongs to PDAAQ/PC-FGF/S cathode when integrated with PC-FGF/TiO_2_/MWCNT-coated separator with initial discharge capacity of 800 mAh g^−1^ at 0.5 C and superior capacity retention of 700 mAh g^−1^ after 100 cycles.

This change in the trend of discharge capacity is related to the effective role of PC-FGF/TiO_2_/MWCNT-coated separator with high-ion selectivity especially in long-life cycling to trap undesired polysulfides species which release from PDAAQ/PC-FGF/S toward the anode and limit diffusion of polysulfides to anode side [35, 36]. As shown in Fig. 3b–d, polarization potential for PDAAQ/PC-FGF, PDAAQ/CTAB/PC-FGF, and PC-GF/TiO_2_/MWCNT are 238, 114, and 327 mV respectively. By incorporation of PDAAQ into PC-FGF and CTAB/PC-FGF coatings, the cells showed higher discharge capacity after 100 cycles and lower polarization potential indicating smaller reaction barrier due to high redox potential and polysulfide adsorption capability of PDAAQ.

**Fig. 4:**
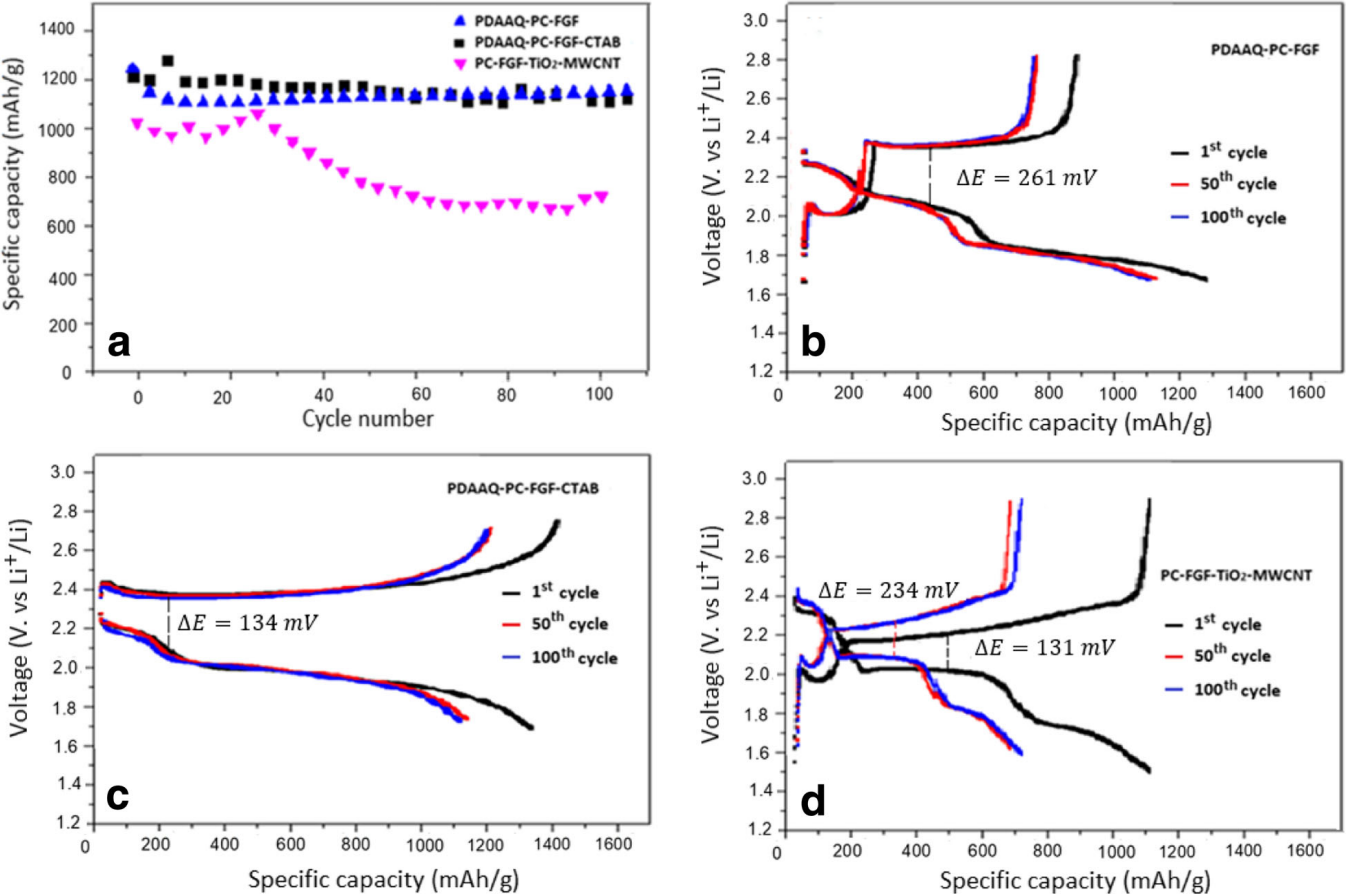
**a** Cycling performance of Li-S cells with TiO_2_/PC-FGF/S cathode and three different coated separators at current density of 0.5 C. **b** Galvanostatic charge/discharge profiles for the 1st, 5th, 100th cycles of Li-S cells with PDAAQ/PC-FGF, **c** PDAAQ/PC-FGF/CTAB, **d** PC-FGF/TiO_2_/MWCNT interlayers at 0.5 C with potential window of 1.5–3 V versus Li^+^/Li^0^

Figure 4a shows the cycling performance of the cells with TiO_2_/PC-FGF/S cathode and the same three separators. The cells with PDAAQ/PC-FGF and PDAAQ/PC-FGF/CTAB-coated separators showed very similar performance with the initial capacities of 1241 and 1232 mAh g^−1^ at 0.5 C, respectively, and retained the capacities at 1100 and 1096 mAh g^−1^ after100 cycles respectively. PDAAQ containing separators exhibited better capacity retention performance after 100 cycles when using TiO_2_/PC-FGF/S cathode, compared to PDAAQ/PC-FGF/S cathode. The cell with PC-FGF/TiO_2_/MWCNT separator and TiO_2_/PC-FGF/S cathode showed the initial capacity of 1011 mAh g^−1^ and capacity retention of capacity of 697 mAh g^−1^ at 0.5 C after 100 cycles. In Fig. 4b–d, charge/discharge voltage profiles for the cells with three different separators are shown. PDAAQ/PC-FGF and PDAAQ/PC-FGF/CTAB-coated separator exhibited low polarization potential of 261 and 134 mV and capacity retention of 1100 and 1096 mAh g^−1^ respectively. Due to electrostatic interaction between Br^−^ ions in CTAB and negatively charged polysulfides species, polarization potential reduced to 134 Mv indicating smaller reaction barrier and higher active material reutilization. For PC-FGF/TiO_2_/MWCNT-coated separator, low polarization potential was reported at the first cycle (131 mV). After 50 cycles, polarization potential increased to 234 mV due to the formation of undesirable polysulfides species which restricted active material reutilization and blocked lithium-ion diffusion (Fig. 4d).

**Fig. 5:**
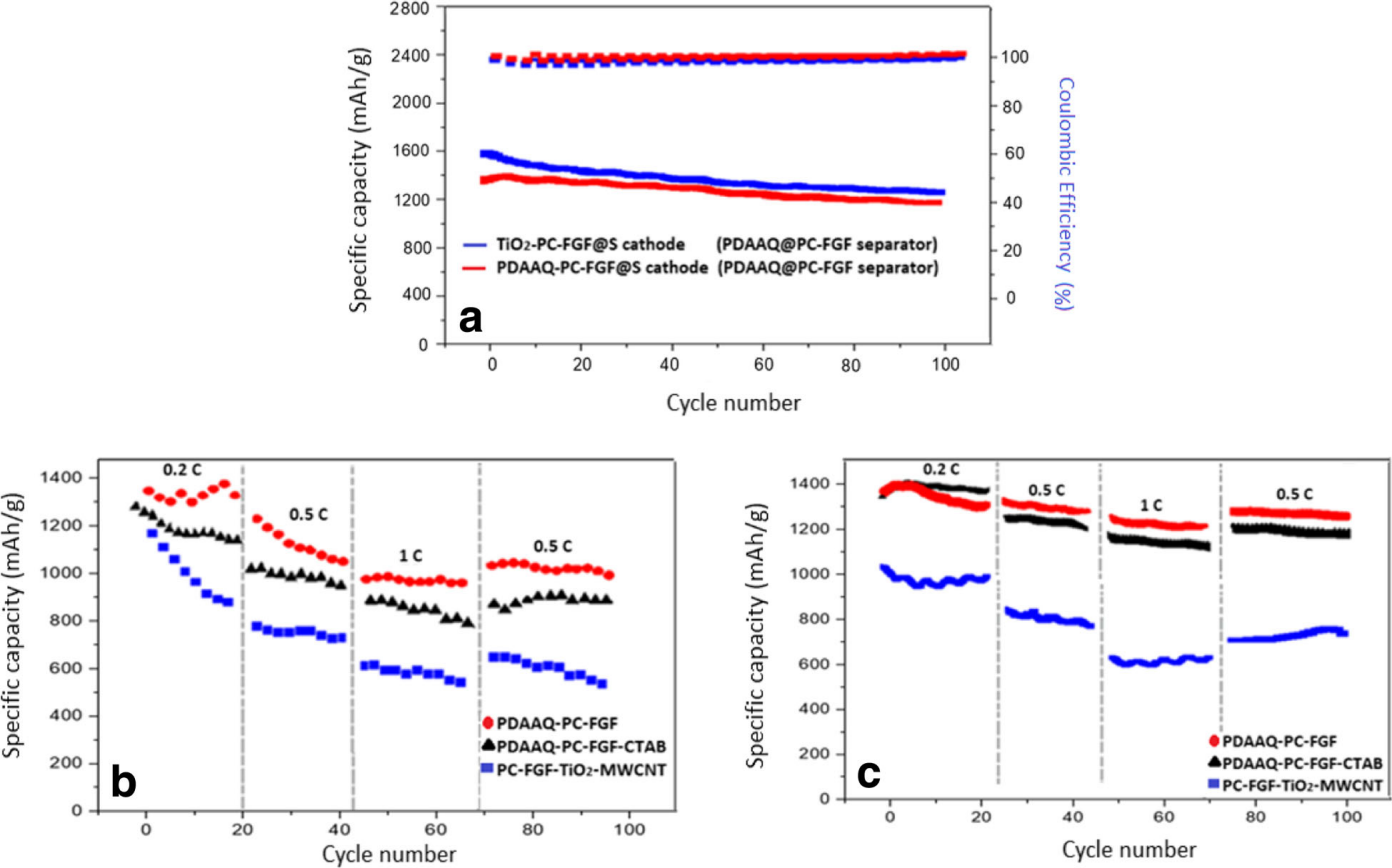
**a** Cycling performances and Coulombic efficiencies of Li-S cells with PDAAQ/PC-FGF separator and two different cathodes at 0.2 C. **b** Rate performance of Li-S cells with PDAAQ/PC-FGF/S cathode and three different separators. **c** Rate performance of Li-S cells with TiO_2_/PC-FGF/S cathode and three different separators

Cycling performances and coulombic efficiencies of the cells with PDAAQ/PC-FGF separator and the two different cathodes were tested and results are shown in Fig. 5a. Coulombic efficiencies are about 100% for the cells with each of the cathodes and PDAAQ/PC-FGF-coated separator. The cell with TiO2/PC-FGF/S cathode retained a capacity of 1381 mAh g^−1^ after 100 cycles at 0.2 C, while the cell with PDAAQ/PC-FGF/S cathode showed the specific capacity of 1243 mAh g^− 1^ after 100 cycles at 0.2 C. TiO_2_/PC-FGF/S cathode could trap negatively charged polysulfides with strong chemical adsorption. Unabsorbed polysulfides during charge process are trapped by PDAAQ/PC-FGF-coated separator through strong binding energy between positively charged NH_2_ in PDAAQ and negatively charged polysulfides. Also, oxygen-containing groups in PDAAQ act as Li-ion hopping site. Besides benefitting from PDAAQ, PC-FGF with a polycarboxylate functionalized electron-donating group increases redox activity in PDAAQ/PC-FGF separator with capacity retention of 85.7% after 100 cycles. PDAAQ/PC-FGF/S cathode functioned as a redox-active cathode which allowed polysulfides to undergo several oxidation and reduction reactions at the cathode. PDAAQ/PC-FGF/S cathode with PC-FGF separator showed capacity retention of 86.4% after 100 cycles. In Fig. 5b, c, rate capability of PDAAQ/PC-FGF/S and TiO_2_/PC-FGF/S cathodes are shown. PDAAQ/PC-FGF/S cathode with three different interlayers were tested at 0.2 C, 0.5 C, 1 C, and back to 0.5 C (Fig. 5b). The cell with PDAAQ/PC-FGF separator showed higher initial capacity of 1370 mAh g^−1^ and the capacity declined to 1263, 1036, and then back to 1091 mAh g^− 1^ respectively. The cell with PDAAQ/PC-FGF/CTAB delivered specific capacities of 1290, 1053, and 947 mAh g^−1^ at 0.2 C, 0.5 C, and 1 C. The capacity retained without significant change at 941 mAh g^−1^ at 0.5 C with retention rate of 72%. For PC-FGF/TiO_2_/MWCNT, separator fast decay of capacity were observed specially during early cycles. It showed an initial capacity of 1190 mAh g^−1^ at 0.2 C but the capacity declined gradually to 805 and 671 mAh g^−1^ at current densities of 0.5 and 1 C, and then at 701 mAh g^−1^ at 0.5 C (Fig. 5b).

Figure 5c shows the rate capability tests for TiO_2_/PC-FGF/S cathode with PDAAQ/PC-FGF, PDAAQ/PC-FGF/CTAB, and TiO_2_/MWCNT/PC-FGF separators at 0.2 C, 0.5 C, and 1 C. The cell with PDAAQ-PC-FGF demonstrated an initial capacity of 1367 mAh g^−1^ and the capacity retained at 1358 and 1298 mAh g^−1^ at 0.5 C and 1 C, and then at 1348 mAh g^−1^ at 0.5 C with a retention rate of 98% after 100 cycles, showing superior stability and very low decay rate. It was found that the electrochemical performance of the cell with PDAAQ/CTAB/PC-FGF separator was similar to that of PDAAQ/PC-FGF separator especially in early cycles. PDAAQ/CTAB/PC-FGF separator delivered initial capacity of 1351 mAh g^−1^ at 0.2 C and the capacity retained at 1273 and 1205 mAh g^−1^ at 0.5 C and 1 C, and then at 1224 mAh g^−1^ at 0.5 C with a retention rate of 90% (Fig. 5c). By adding CTAB to separator coating more polysulfides are pushed toward the cathode, and as the number of cycles increased the more polysulfides are accumulated on the cathode surface, subsequently the less polysulfides could be reutilized by TiO_2_ nanoparticles containing cathode. This result proves that CTAB addition to coating has adverse effect as the number of cycles increase. TiO_2_/MWCNT/PC-FGF separator exhibited unstable and fast declining for specific capacity at various current densities. At 0.2 C, 0.5 C, and 1 C capacity retained at 1048, 881, and 691 mAh g^−1^ respectively and when current density returned back to 0.5 C, specific capacity of 738 mAh g^−1^ can be obtained. Capacity retention was just delivered 70% (Fig. 5c).

**Fig. 6:**
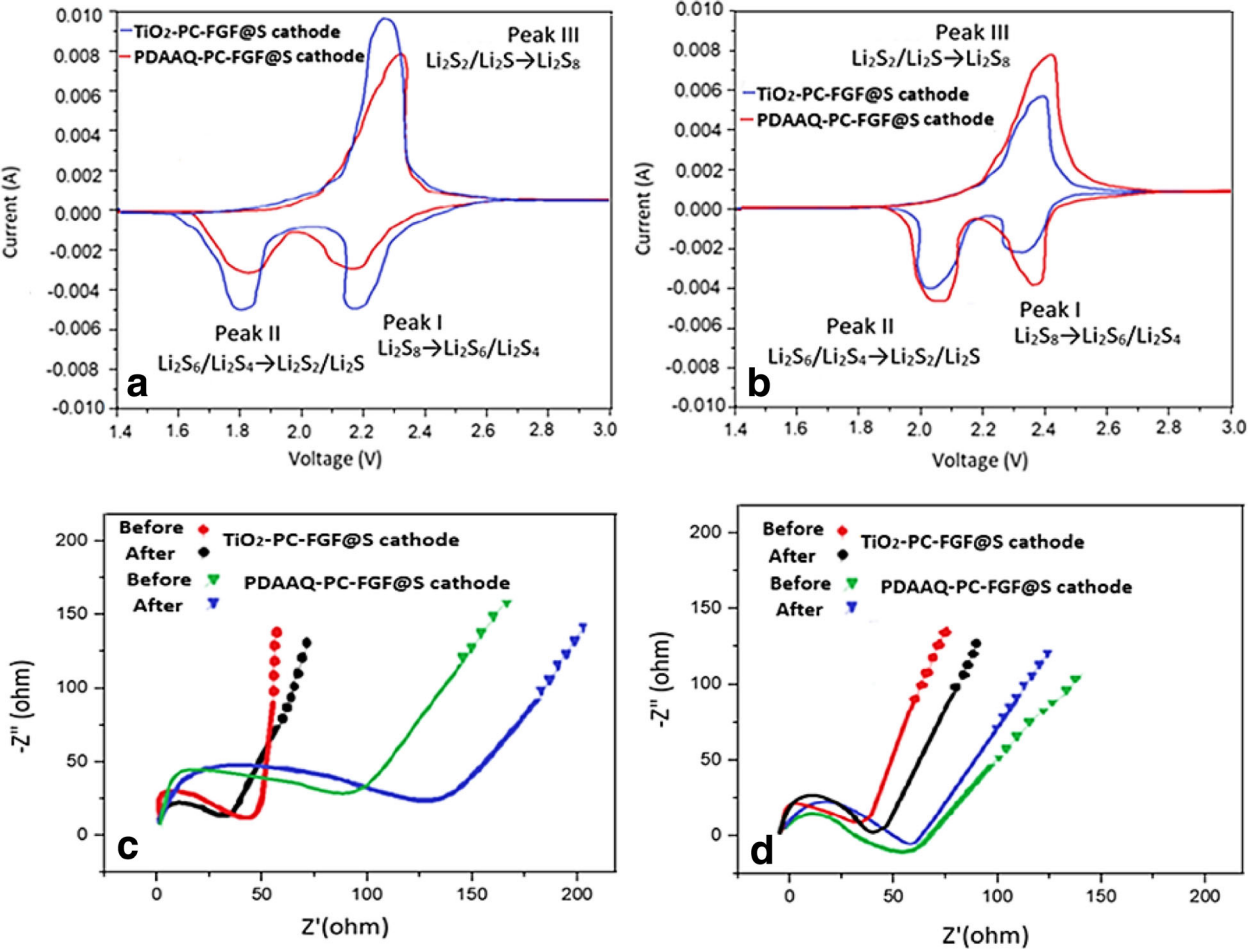
CV curves of Li-S cells at scan rate of 0.2 mV S^−1^ **a** with PDAAQ/PC-FGF separator, **b** with PDAAQ/PC-FGF/CTAB separator, **c** electrochemical impedance spectrum (EIS) curves of PDAAQ/PC-FGF separator with two different cathodes 1st and 100th cycles at 0.5 C, **d** EIS curves of PDAAQ/PC-FGF/CTAB separator with two different cathodes 1st and 100th cycles at 0.5 C

Figure 6a shows the cyclic voltammetry (CV) in the voltage range of 1.4–3 V (vs. Li/Li+) at a scanning rate of 0.2 mV s^−1^ for PDAAQ/PC-FGF/S and TiO_2_/PC-FGF/S cathode when glass fiber separator was coated with PDAAQ-PC-FGF. In the voltage range of 1.4–3 V, TiO_2_/PC-FGF/S and PDAAQ/PC-FGF/S cathode reveal 2.20, 1.80, and 2.19, 1.83 V cathodic peaks respectively which display the formation of long-chain lithium polysulfide (Li_2_S_n_) and further reduction to Li_2_S_2_ or Li_2_S. One anodic peak at 2.30 and 2.36 V for TiO_2_/PC-FGF/S and PDAAQ/PC-FGF/S cathode reveals oxidation of Li_2_S_2_ or Li_2_S to long-chain lithium polysulfide. CV of PDAAQ/PC-FGF/S and TiO_2_/PC-FGF/S cathode with PDAAQ/PC-FGF/CTAB separator was investigated in Fig. 6b. Two cathodic peaks are located at 2.28 and 2.0 V for TiO_2_-PC-FGF-S cathode and 2.33 and 2.02 V for PDAAQ/PC-FGF/S cathode implying larger electrochemical polarization in comparison with PDAAQ/PC-FGF separator as shown in Fig. 6a. As indicated in Fig. 6a, TiO_2_/PC-FGF/S in PDAAQ/PC-FGF separator display smaller voltage polarization with more positive anodic peak and more negative cathodic peaks contribute to accelerate redox kinetic reactions between polysulfide intermediate products and TiO_2_ nanoparticles in the cathode. The same improvement in electrochemical polarization has been observed for CTAB-based separator when the cell is assembled with PDAAQ/PC-FGF/S cathode (Fig. 6b).

As indicated in Fig. 6a, b, peak І displays conversion reactions from elemental sulfur to long-chain polysulfides. Peak ІІ identifies the conversion of soluble polysulfides to short-chain Li_2_S_2_/Li_2_S polysulfides. Converting short-chain back to long-chain polysulfides and further to sulfur is shown by peak ІІІ.

The Nyquist plots for two different cathodes before and after 100 cycles are shown in Fig. 6c, d. The semicircle at high frequencies represents the resistance of the surface films and at medium frequencies displays the charge transfer resistance at the electrode−electrolyte interface. TiO_2_/PC-FGF/S cathode with PDAAQ/PC-FGF separator displays smaller charge transfer resistance before and after 100 cycles in compare to PDAAQ/PC-FGF/S cathode with the same separator. The reason behind that is referred to high porosity of TiO_2_ nanoparticles, electrolyte could permeate faster, suggesting faster ion diffusion and better interfacial property (Fig. 6c). Figure 6d exhibits TiO_2_/PC-FGF/S and PDAAQ/PC-FGF/S cathode with PDAAQ/CTAB/PC-FGF-coated separator before and after 100 cycles. For TiO_2_/PC-FGF/S cathode displays smaller interfacial resistance than those of PDAAQ/PC-FGF/S cathode and better electrical conduction for charge transfer [37].

In PDAAQ/PC-FGF/S cathode bigger diameter of the semicircle in the Nyquist plot reflects the accumulation of polysulfides which limit electrolyte infiltration and decrease electrochemical performance of the cell. As shown in Fig. 6c, d, the resistance of all the cells after cycling were more compared with before cycling resistance. Just for PDAAQ/PC-FGF/S cathode with PC-FGF/PDAAQ/CTAB separator, the value of charge transfer resistance after cycling decreases. In this case, electrolyte infiltration improved after 100 cycles led to fast charge transport and fewer degree of polysulfide diffusion [38, 39].

The above-mentioned results proved that the cell with TiO_2_/PC-FGF/S cathode and PDAAQ/PC-FGF, PDAAQ/PC-FGF/CTAB-coated separators showed the best cycling performance and the lowest interfacial resistance among the others. To confirm the efficiency of our suggested cathode and separator design to hinder polysulfide diffusion, the Li-S cells were disassembled after 100 cycles at 0.5 C, and additional inspection into the lithium polysulfide deposition on the separator surfaces (cathode-facing side) is performed by SEM images and related sulfur mappings.

**Fig. 7:**
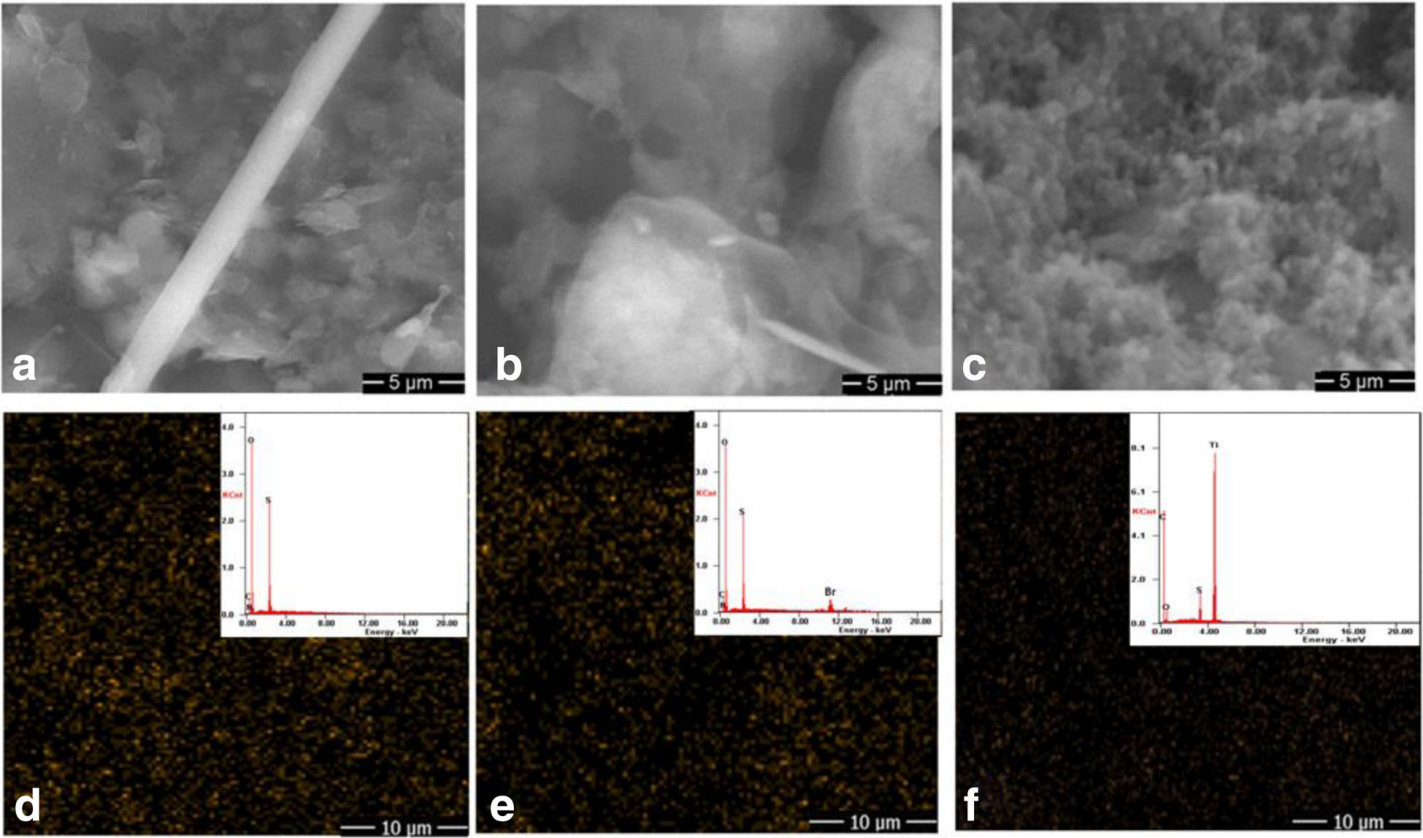
SEM images of the surface view of **a** PDAAQ/PC-FCF **b** PDAAQ/PC-FCF/CTAB **c** PC-FGF/TiO_2_/MWCNT coated separators cycled for 100 cycles at 0.5 C with TiO_2_/PC-FGF/S cathode, EDS mapping of sulfur element on the separator surface cycled **d** with PDAAQ/PC-FCF **e** PDAAQ/PC-FCF/CTAB, and **f** PC-FGF/TiO_2_/MWCNT-coated separators with TiO_2_/PC-FGF/S cathode; the insets are the related elemental spectra

Figure 7a–c displays SEM images of the separator surfaces after 100 cycling with three different coatings. As shown in Fig. 7a, b, polysulfides were deposited around PDAAQ at PDAAQ/PC-FGF and PDAAQ/PC-FGF/CTAB coated separators when integrated with TiO_2_/PC-FGF/S cathode which means that it could restrain the polysulfide migration toward the anode and improve cell performance. Also, results are in good agreement with the sulfur-mapping analysis by EDS. As shown in Fig. 7d, e, sulfur distribute homogeneously in the PDAAQ/PC-FGF and PDAAQ/PC-FGF/CTAB-coated separators, which proved superior adsorption and permeation ability of coated glass fibers. The stronger sulfur signals in the PDAAQ/PC-FGF and PDAAQ/PC-FGF/CTAB-coated separators implies high capability of PDAAQ-based separators to trap polysulfides when integrated with TiO_2_/PC-FGF/S cathode.

EDS mapping showed high and homogenous distribution of sulfur when PDAAQ/PC-FGF-coated separator was introduced and functioned as an efficient interlayer to suppress movement of Li_2_S_2_ and Li_2_S polysulfides toward the anode. It could alleviate shuttle effect through its superior ability to adsorb short-chain polysulfides and enhance active material reutilization. PDAAQ/PC-FGF/CTAB-coated separator showed similar behavior with weaker sulfur intensity in the cathode-facing side of the separator. PC-FGF/TiO_2_/MWCNT-coated separator displayed the lowest sulfur intensity and sulfur adsorption on the separator surface among others (Fig. 7f), verifying less polysulfides could be trapped by the separator leading to loss of active material and high capacity fading rate.

## Conclusions

TiO_2_/PC-FGF and PDAAQ/PC-FGF incorporated S cathodes were prepared through one step slurry method, and the cells with these cathodes and three different coated separators were tested and their electrochemical performances were compared. PDAAQ/PC-FGF and PDAAQ/PC-FGF/CTAB-coated separators show superior capacity retention and high cyclability for both cathodes offering high material reutilization. The most promising result belongs to TiO_2_/PC-FGF/S cathode with PDAAQ/PC-FGF separator which minimizes diffusion of polysulfides through the cell and reduce the charge transfer resistance. The polar nature of the PC-FGF cathode as well as highly porous structure of TiO_2_ provides both physical confinement and chemical interaction with polysulfides. The incorporation of polymeric compounds and TiO_2_ has also been proposed in the separator to enhance active material reutilization. Further research in Li − S batteries should be investigated not only demonstrates an effective cathode material, but also indicates the importance of separatror materials on battery performance.

## Data Availability

All data used within this manuscript is available upon request.

## Abbreviations

CTAB: Cetyltrimethylammonium bromide
CV: Cyclic voltammogram
DME: 1,2-dimethoxyethane
DOL: 1,3-dioxolane
LiNO_3_: Lithium nitrite
LiTFSI: Lithium bis (trifluoromethane) sulfonamide lithium
NMP: N-methyl-2 pyrrolidone
PC-FGF: Polycarboxylate functionalized graphene
PDAAQ: 1, 5-Poly diaminoanthraquinone
PVDF: Polyvinylidene fluoride
SEM: Scanning electron microscopy
TiO_2_: Titanium dioxide
